# Development of a Sensitive Bioassay for the Analysis of IGF-Related Activation of AKT/mTOR Signaling in Biological Matrices

**DOI:** 10.3390/cells10030482

**Published:** 2021-02-24

**Authors:** Michael Walz, Christine Höflich, Christina Walz, Daniela Ohde, Julia Brenmoehl, Mandy Sawitzky, Andreas Vernunft, Uwe K. Zettl, Susanne Holtze, Thomas B. Hildebrandt, Eckhard Wolf, Andreas Hoeflich

**Affiliations:** 1 Institute of Genome Biology, Leibniz Institute for Farm Animal Biology (FBN), Wilhelm-Stahl Allee 2, 18196 Dummerstorf, Germany; walz.michael@fbn-dummerstorf.de (M.W.); walz@fbn-dummerstorf.de (C.W.); ohde@fbn-dummerstorf.de (D.O.); brenmoehl@fbn-dummerstorf.de (J.B.); mandysawitzky@gmx.de (M.S.); 2 Ligandis Biomarker Diagnostics, Dorfstr. 14, 18276 Gülzow-Prüzen, Germany; christine.hoeflich@ligandis.de; 3 Institute of Reproductive Biology, Leibniz Institute for Farm Animal Biology (FBN), Wilhelm-Stahl Allee 2, 18196 Dummerstorf, Germany; vernunft@fbn-dummerstorf.de; 4 Department of Neurology, Neuroimmunological Section, University Medicine Rostock, Gehlsheimer Str. 20, 18147 Rostock, Germany; uwe.zettl@med.uni-rostock.de; 5 Department of Reproduction Management, Leibniz-Institute for Zoo and Wildlife Research, Alfred-Kowalke-Str. 17, 10315 Berlin, Germany; holtze@izw-berlin.de (S.H.); hildebrand@izw-berlin.de (T.B.H.); 6 Chair of Molecular Animal Breeding and Biotechnology and Laboratory for Functional Genome Analysis (LAFUGA), Gene Center, LMU Munich, Feodor-Lynen-Str. 25, 81377 Munich, Germany; ewolf@genzentrum.lmu.de

**Keywords:** IGF, bioactivity, bioassay, mTOR, AKT phosphorylation, serum, cerebrospinal fluid, milk, colostrum, western immunoblotting, capillary immuno-electrophoresis, BIRA assay

## Abstract

The bioactivity of the IGF system is not a function of isolated hormone concentrations in a given biological matrix. Instead, the biological activities of IGFs are regulated by IGFBPs, IGFBP proteases, and inhibitors of IGFBP proteases. Therefore, assays based on IGF-related bioactivity may describe functions of the complete IGF system in a given biological matrix. Of particular interest are the IGF system effects on the AKT/mTOR pathway, as a dominant system for controlling growth, metabolism, and aging. In order to improve the sensitivity of IGF-dependent bioactivity, we made use of the known short-term and enhancing effects of IGFBP2 on the intracellular PI3K pathway. As a specific readout of this pathway, and further as a marker of the mTOR pathway, we assessed the phosphorylation of AKT-Ser473. Preincubation using IGFBP2 enhanced IGF1-dependent AKT-Ser473 phosphorylation in our experimental system. The assay’s specificity was demonstrated by inhibition of IGF1 receptors outside or inside the cell, using antiserum or small molecule inhibitors, which reduced AKT phosphorylation in response to exogenous IGF1 (*p* < 0.05). The maximal response of AKT phosphorylation was recorded 15 to 60 min after the addition of IGF1 to cell monolayers (*p* < 0.001). In our cellular system, insulin induced AKT phosphorylation only at supra-physiological concentrations (µM). Using this novel assay, we identified the differential biological activity of the IGF system in AKT-Ser473 phosphorylation in serum (mouse, naked mole rat, and human), in cerebrospinal fluid (human), and in colostrum or mature milk samples (dairy cow). We have developed a sensitive and robust bioassay to assess the IGF-related activation of the AKT/mTOR pathway. The assay works efficiently and does not require expensive cell culture systems. By using capillary immuno-electrophoresis, the readout of IGF-related bioactivity is substantially accelerated, requiring a minimum of hands-on time. Importantly, the assay system is useful for studying IGF-related activity in the AKT/mTOR pathway in a broad range of biological matrices.

## 1. Introduction

**Figure 1:**
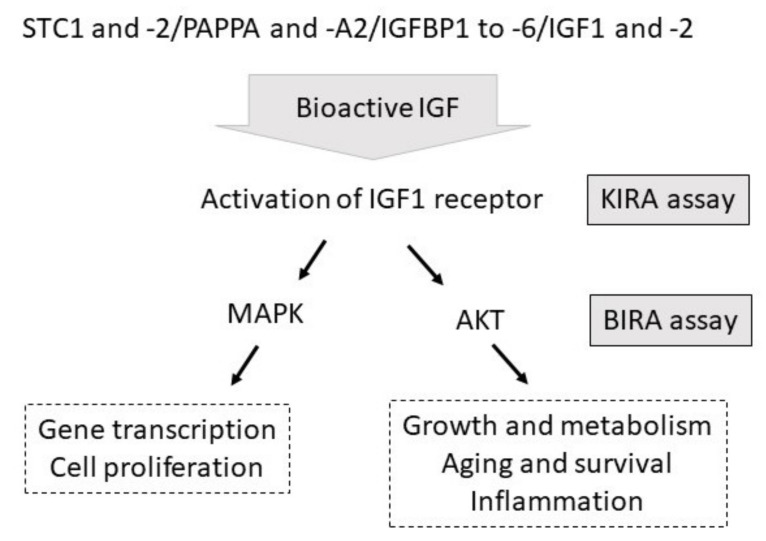
Control of IGF-related bioactivity by inhibitors (STC1 and STC2), proteases (PAPPA and PAPPA2), and IGFBPs (IGFBP1 to 6). IGF-dependent activation of the IGF1 receptor is quantified by the KIRA assay. IGF-related activation of AKT can be assessed by the novel BIRA assay (STC1 and 2: stanniocalcin 1 and 2; PAPPA and A2: pregnancy-associated protein protease A and A2; IGF: insulin-like growth factor; IGFBP: IGF-binding protein; MAPK: mitogen-activated protein kinase; AKT: protein kinase B; KIRA: **ki**nase **r**eceptor **a**ctivation; BIRA: IGF**B**P2-enhanced **I**GF-*r*elated **A**KT activation).

Knowledge of the insulin-like growth factor (IGF) system has increased recently. It is realized now that control of IGF functions by proteases for IGF binding proteins (IGFBP) and inhibitors of IGFBP proteases is a central part of the physiological regulation of growth and development in vivo and in vitro [1], rather than being a rare event in certain malignancies or during pregnancy. Under this current concept of the IGF system, it is also clearer now that quantification of total or free IGFs, IGFBPs, IGFBP proteases, or IGFBP inhibitors only represents an assessment of surrogate biomarkers for the description of the function of the IGF system. Instead, functional assays of IGF-related bioactivity are required to integrate the growing number of IGF system effectors. Classically, for the readout of IGF-related bioactivity, assays of cell proliferation [2], colony formation [3], or cell migration [4] have been performed in the past, using formulations of recombinant IGF1 or IGF2. In addition, assays of the analysis of IGF-related bioactivity on the level of IGF1 receptor phosphorylation (kinase receptor activation, KIRA assay) have been developed [5,6]. The KIRA assay has been successfully applied for the study of IGF-related activity in different biological matrices, including serum [7], ascites [8], as well as pleural [9] and cerebrospinal fluids [10]. KIRA assays can provide specific information on the activation of the IGF1 receptor by a given matrix. Specifically, an antiphosphotyrosine antibody is used to detect tyrosine phosphorylation in immobilized IGF1 receptor moieties in response to IGF-related stimulation in vitro [6]. In response to IGF binding to IGF1 receptors, tyrosine phosphorylation provides direct information on IGF1 accessibility for the IGF1 receptors. After binding to IGF1 receptors, IGF1 may induce different signaling cascades, including mitogen-activated protein kinases (MAPK) and phosphatidylinositol 3 kinase (PI3K) or protein kinase B (AKT). Accordingly, the KIRA assay represents an elegant method to identify IGF-related activity at the level of the IGF1 receptor (Figure 1), but does not provide information on the translation of IGF-related bioactivity to distinct signaling cascades within the cell. Another deficit of the KIRA assay is that this assay cannot directly be used to study the biological activity of the IGF system in different cell types, because the enhancement of the signal is dependent on IGF1 receptor-transfected cells.

To analyze the activation of the mammalian target of Rapamycine (mTOR) and AKT and to establish a more flexible cell culture system in which IGF-related bioactivity can be tested in different cell types without the need of transfection, we developed the *BP2-enhanced IGF-related AKT phosphorylation* (BIRA) assay, using IGFBP2 to enhance IGF-related AKT-Ser473 phosphorylation. The mechanism for increasing phosphorylated AKT at serine 473 (AKT-Ser473) by IGFBP2 was provided by Shen et al. [11,12]. Accordingly, membrane-bound IGFBP2 coordinates with IGF1 to induce intracellular signal transduction [12]. The positive effects of IGFBP2 on AKT phosphorylation include inactivation of receptor tyrosine phosphatase beta (RPTPβ) activity by bound IGFBP2, which results in the inhibition of RPTPβ-dependent dephosphorylation of phosphatase and tensin homolog (PTEN). Since dephosphorylated PTEN is required for dephosphorylation of phosphatidylinositol (3,4,5)tri-phosphate (PIP3), activation of AKT-Ser473 is increased.

In our study, we characterize the sensitivity, specificity, and kinetics of the novel assay system and provide a detailed protocol for the application of the BIRA assay in different cell types. Finally, we analyze four different matrices from four different species to provide examples of application, thereby demonstrating the flexibility of the novel BIRA assay. 

## 2. Materials and Methods

### 2.1. Cell Culture

HEK293-10 and HEK293-2 cells originate from the human embryonic kidney fibroblast cell line HEK293, obtained from the American Type Culture Collection (ATCC, Rockville, MD, USA). HEK293 cells were transfected with 8 µg pCMV-int-mIGFBP2 (XhoI linearized) and 0.8 µg EcoRI linearized neomycin resistance plasmid pSV2neo (Clontech, Heidelberg, Germany) [13]. While the HEK293-10 cell clone is characterized by high secretion of IGFBP2 in the presence or absence of neomycin, the HEK293-2 cell clone also grows in the presence of neomycin, however without production or secretion of IGFBP2. HuH-7, C2C12, and 3T3-L1 cells were also obtained from ATCC (Rockville, MD, USA). All cell lines were cultured in cell culture dishes measuring 100 mm in diameter (#83.3902, Sarstedt AG and Co. KG, Nümbrecht, Germany) in cell culture media (for specific media see Table 1) with 10% fetal bovine serum (FBS; 10500-064; Gibco, Carlsbad, CA, USA) and 1% 100× penicillin/streptomycin/amphotericin B mixture (882087; Lonza, Gampel, Switzerland)), incubated at 37 °C and 5% CO_2_. The media were renewed every 2–3 days.

After 6–7 days, the cells were sub-cultivated. Therefore, the cell culture medium was removed, the monolayers were rinsed in phosphate-buffered saline (PBS, 137 mM NaCl, 2.7 mM KCl, 10 mM Na_2_HPO_4_, 1.8 mM KH_2_PO_4_), and 3 mL PBS with 0.25% trypsin and 0.53 mM EDTA (10× Trypsin/EDTA-Mix, BE02-007E, Lonza) was added. After incubation at 37 °C for 10 min, the cells were detached and the reaction was stopped by adding 6 mL cell culture medium. The cells were centrifuged at 800 RCF for 10 min at room temperature, and after aspiration of the supernatant, the pellet was resuspended in fresh cell culture medium. The sub-cultivating ratio was 1:10 for HEK293-2, HEK293-10, and 3T3-L1 cells, and 1:50 for the cell lines HuH-7 and C2C1. For all experiments, the cells were seeded in 24-well-plates with 10^5^ cells per well in 500 µL cell culture medium, and every two to three days the medium was renewed (Eagle’s minimal essential medium—EMEM; Dulbeco’s modified Eagle’s medium—DMEM).

### 2.2. Serum, Cerebrospinal Fluid, and Milk Samples

Serum samples from two different mouse lines (DU6 and DUC) were collected previously [14] at the Leibniz Institute for Farm Animal Biology (FBN). DU6 is a mouse line selected for high male body mass at the age of 42 days. This mouse line was developed from the same genetic background, which is also present in the second mouse line used in this study, representing the unselected non-inbred DUC control mouse line. Serum samples were collected from male mice sacrificed at the age of 28, 49, 77, and 112 days [14], which were stored at −20 °C. The absolute serum IGF1 concentrations were published in a recent study [15]. The animal experiments were performed according to national and international guidelines and approved by the institutional (Animal Protection Board from the Leibniz Institute for Farm Animal Biology) and national (Animal Protection Board Mecklenburg-Vorpommern) protection boards (file number: LALLF M-V/TSD/7221.3-1.2-037/06). For this study, serum samples from five animals of each age group and a selection line were pooled and diluted to 1:5 in PBS. 

Serum samples from naked mole rats were obtained in accordance with national and institutional animal care guidelines and approved by the ethics committee of the State Office for Health and Social Affairs, Berlin, Germany (#ZH 156, G02217/12, T 0073/15) from animals kept at the Leibniz Institute for Zoo and Wildlife Research (IZW). Serum samples were stored in liquid nitrogen until further use in this study and were derived from four female naked mole rats (three workers at ages of 0.9, 1.0, and 1.6 years, and one queen at the age of 3.5 years).

Human serum and human cerebrospinal fluid (hCSF) samples were collected at the Department of Neurology from the University Medical Center in Rostock, Germany. The samples were from multiple sclerosis patients undergoing a triamcinolone therapy between 2009 and 2012 and stored at −80 °C before use [16]. Concentrations of IGFs and IGFBPs in serum and CSF were published recently [17]. The use of all samples was approved by the ethics committee of the University Medical Center Rostock (approval A 2016-0088). For the experiment, matched samples of human serum and hCSF were pooled from 10 patients. 

The milk samples were collected during regular milking from ten multiparous Holstein–Friesian (HF) dairy cows housed in the Experimental Animal Facility for Cattle of the FBN. The milk samples were collected at eight time points from all animals during pregnancy (around d40, d135, and d220 postconceptional), around calving (colostrum d0, d1, and d2 postpartum), and around day 7 and 30 postpartum of the following lactation period. Sampling was performed by the Institute of Reproductive Biology, FBN, between 2017 and 2018 and samples were stored at −20 °C before use. For acidification, 1 mL of each milk sample was supplemented with 100 µL 1M hydrochloric acid, incubated for 10 min at 37 °C, and then centrifuged at 16,800 RCF for 5 min. After acidic precipitation of caseins and centrifugation, the supernatants were used for further analysis.

### 2.3. Preincubation of Cell Monolayers in IGFBP2 Containing Cell Culture Medium

At the point of confluence in 24-well plates, the cells were used for the bioassay (up to 10 days after seeding). Cells were exposed to medium containing only 0.5% FBS in the presence or absence of human recombinant IGFBP2 (RD172583100, BioVendor GmbH, Kassel, Germany) for 24 h. IGFBP2 was tested at different concentrations (33.75, 67.5, 125, 250, 500, 1000, and 2000 ng/mL).

### 2.4. Preincubation of Cell Monolayers with Inhibitors of IGF1/Insulin Signaling

To study the specific cell response to IGF1, inhibitors of the IGF1 receptor were added to the cells two hours before exposure to the samples. The antibody αIR3 (Cat. No. GTX16890, GeneTex Inc., distributed by Biozol, Eching, Germany) was used at concentrations of 0.1 mg/L and 1 mg/L in EMEM. The small molecule inhibitor BMS-754807 (Cat. No. BM0003-5MG, Sigma-Aldrich, Taufkirchen, Germany) was dissolved in dimethyl sulfoxide—DMSO; D8418, Sigma-Aldrich) to create a 390.65 M stock solution and then dissolved in EMEM for the final test solutions at concentrations of 0.01 µM and 1 µM. For the inhibitor experiments with BMS-754807, the control contained 0.1% DMSO in EMEM. The inhibitors and the corresponding controls were incubated for 2 h and subsequently discarded before the cells were exposed to biological matrices or serial hormone dilutions. 

### 2.5. Bioassay and Lysis

The samples were either taken directly (milk samples and CSF) or diluted in PBS (20% serum in PBS). In addition, serial dilutions of human recombinant IGF1 (Cat. #100-11, PeproTech, Inc., Rocky Hill, NJ, USA) or insulin (#I3536, Sigma Aldrich, Darmstadt, Germany) in PBS were used.

After discarding the 0.5% FBS cell culture medium or inhibition medium, 200 µL of test medium was added to each well, which were then incubated for a set time frame (20 min, if not stated otherwise) at 37 °C. The test medium was then discarded and the cells were washed in PBS before cell lysis in 100 µL lysis buffer (1.5 tablets of complete mini protease inhibitor; Hoffmann-La Roche, Basel, Switzerland), 31.25 mM tris(hydroxymethyl)-aminomethane, 1% sodium dodecyl sulfate (SDS), 5% glycerine in 17 mL water, and 1.5 mL 10x lysis buffer (Cell Lysis Buffer, Cell Signaling Technologies, Danvers, MA, USA). Five minutes later, the lysates were collected, homogenized, and denatured for 5 min at 95 °C.

### 2.6. Protein Concentration

The protein concentration was assessed using bicinchoninic acid (BCA1-1KT, Merck, manufacturer’s protocol for microtiter assay) and calculated in GraphPad Prism 9. For the final protein concentration of 1 µg/mL, the samples were diluted to 1x Laemmli (31.25 mM tris(hydroxymethyl)-aminomethane, 1% sodium dodecylsulfate (SDS), 5% glycerine) dyed with 0.01% bromophenol blue. Βeta-mercaptoethanol was added to a final concentration of 0.4%.

### 2.7. Electrophoresis and Western Immunoblotting

For electrophoresis, Bio-Rad TGX Stain-Free FastCast Acrylamide-Kit (Bio-Rad Laboratories GmbH, Munich, Germany) gels were used. All gels were blotted on polyvinylidene fluoride membranes (PVDF, pore size 0.45 µm, Carl Roth GmbH + Co. KG, Karlsruhe, Germany), blocked for 1 h with 3% powdered milk in PBS and incubated overnight with a primary antibody for AKT phosphorylated at serine 473 (CST #9271, dilution 1:1000, Cell Signaling Technologies). After washing, the membranes were incubated for 2 h with a secondary antibody (antirabbit IgG HRP, CST #7074, dilution 1:2000, Cell Signaling Technologies). Bands were visualized using Lumigen ECL Ultra (Lumigen Inc., Southfield, MI, USA) in a Bio-Rad Chemi-Doc MP system (Bio-Rad Laboratories GmbH). The images were analyzed using Image Lab Ver. 6.0.1 software (Bio-Rad Laboratories GmbH, Hercules, CA, USA) and normalized for total protein concentration. The molecular weight of phosphorylated human AKT is predicted to be 56–57 kDa. 

### 2.8. Capillary Immuno-Electrophoresis (WES^TM^)

The analysis with the WES device from Protein Simple (San Jose, CA, USA) was performed according to the manufacturer’s manual with a WES separation kit for 12–230 kDa with 8 × 25 capillary cartridges (#SM-W004-1), the affiliated standard pack (#PS-ST05-8), an antirabbit detection module (DM-001), and the same primary antibody used for Western immunoblotting (phos-AKT, CST #9271, dilution 1:50). All devices and chemicals, except the primary antibody, were purchased from Protein Simple. The analysis was performed with the software Compass for SW (Protein Simple). The settings for the specific analysis are provided in the Supplementary Information (Table S1).

### 2.9. Statistical Analysis and Graphs

The statistical analysis and the graphs were performed using ANOVA in GraphPad Prism 9 (version 9.0.0). For approximation of the saturation curve for the concentration dependency, the function “specific binding with Hill slope” in GraphPad Prism 9 was used. It has the form $y\left(c\right)=B_{max}\cdot \frac{c^{h}}{ec_{50}{}^{h}+c^{h}}\;$ (*c* = IGF1 concentration, *B_max_* = maximum ligand binding in units of y, *h* = Hill slope, *ec*_50_ = approximated IGF1 concentration with 50% ligand binding).

For the time-dependent response of AKT phosphorylation to a constant IGF1 addition, the function “association then dissociation” in GraphPad Prism 9 was used. It has the following form (Equation (1)). For the regression, the highest mean value of the phos-AKT signal was set and the associated time point to the strongest signal was set as *t*_0_ (*t* = time after IGF1 was added to cells, *B_max_* = maximum ligand binding in units of y, *K_on_* = association constant in min^−1^, *K_off_* = dissociation constant in min^−1^, *t*_0_ = time dissociation is initiated, *HoTNM* = constraint of the ligand in nM).

(1) $$y\left(t\right)=\left\{\begin{array}{c} Eq\cdot (1-\exp\left(-K_{ob}\cdot t\right)\;+\;1,\;\;t<t_{o}\\ Eq\cdot (1-exp\left(-K_{ob}\cdot t_{0}\right)\cdot \exp(K_{off}\left(t-t_{0}\right)\;+\;1,\;\;t\geq t_{0} \end{array}\right.\;\mathrm{with}\;Eq=B_{max}\cdot \frac{ligand}{ligand\;+\;\frac{K_{on}}{K_{off}}}\;\mathrm{and}\;K_{ob}=ligand\cdot \frac{K_{on}}{K_{off}}\;\;\mathrm{with}\;ligand\;=\;HoTNM\cdot 10^{-9}$$

## 3. Results

The aim of this study was to develop a functional bioassay for the study of IGF-dependent activation of the AKT/mTOR pathway.

### 3.1. A Sensitive Assay System for the Study of IGF-Dependent Activation of AKT

Before different biological matrices were tested on HEK293-10 cell monolayers, the BIRA assay was defined and characterized with respect to dose dependency, specificity, and the temporal response of IGF-dependent phosphorylation of AKT. 

#### 3.1.1. Dose-Dependent Increase of AKT Phosphorylation by Human Recombinant IGF1

**Figure 2:**
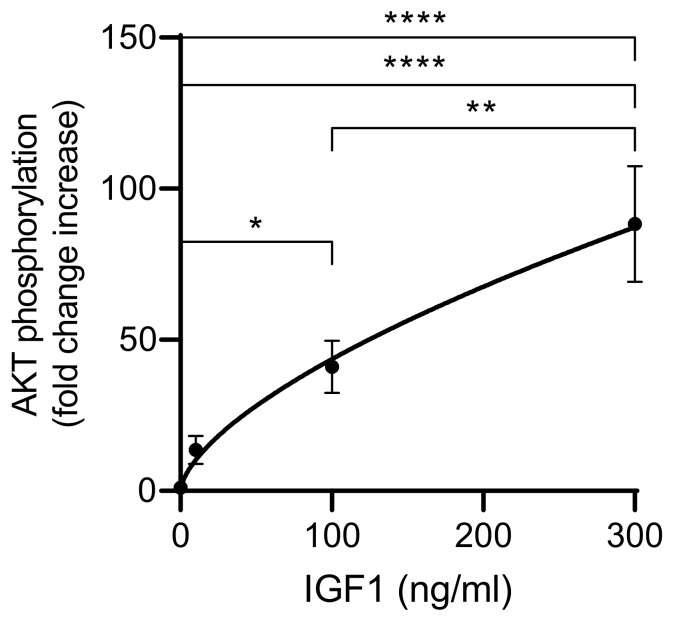
Dose-dependent increase of AKT phosphorylation in IGFBP2-transfected HEK293-10 cells. Cells were incubated in different concentrations of human recombinant IGF1 (0, 10, 100, 300 ng/mL) for 20 min before they were lysed and assayed for phosphorylation of AKT by Western immunoblotting, as described in Materials and Methods (mean ± SEM; *n* = 23, * *p* < 0.05, ** *p* < 0.01, **** *p* < 0.0001; abbreviations are explained in Figure 1).

In the pilot experiment, IGFBP2-transfected HEK293-10 cells secreting high amounts of IGFBP2 and untransfected HEK293 cells were used. Both cell lines were incubated for 20 min in different doses of human recombinant IGF1 before cells were harvested and tested for the levels of phosphorylated AKT (Figure S1). Under the experimental conditions, the addition of IGF1 had a much stronger effect in HEK293-10 cells compared to untransfected HEK293 cells. This effect was assessed by the analysis of AKT phosphorylation using Western immunoblotting or capillary immune-electrophoresis (WES). The dose dependency of AKT phosphorylation in response to IGF1 supplementation was then studied in more detail in HEK293-10 cells only (Figure 2). These cells were highly responsive to the effects of IGF1 regarding phosphorylation of AKT. Incubation in 100 ng/mL for 20 min was sufficient for a significant increase in phosphorylated AKT (p < 0.05). A further increase of the IGF1 concentration resulted in an additional increase of AKT phosphorylation (p < 0.01). Insulin also stimulated phosphorylation of AKT, however only at supraphysiological concentrations (data not shown).

#### 3.1.2. Inhibition of IGF-Dependent AKT Phosphorylation by Antiserum and Small Molecules

**Figure 3:**
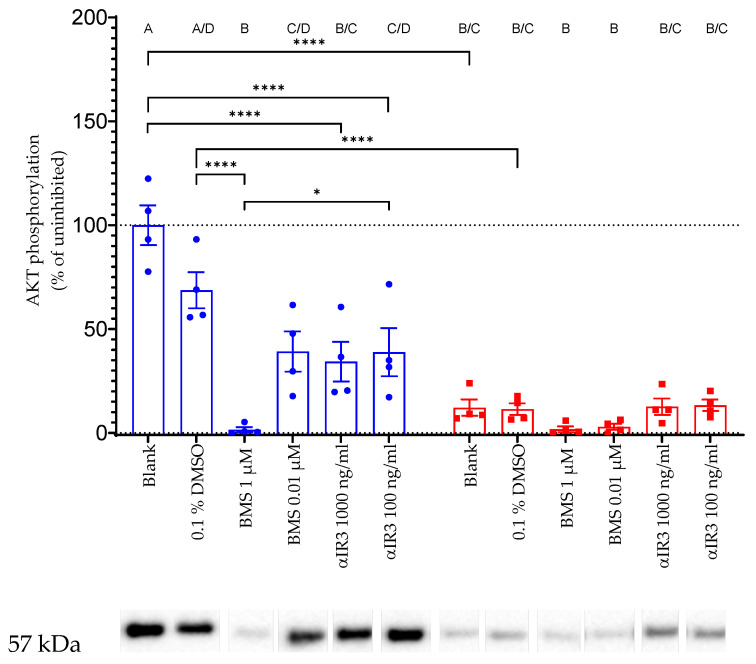
Inhibition of IGF-dependent AKT phosphorylation by IGF1 receptor antiserum (αIR3) and by the small molecule inhibitor BMS-754807 (BMS). HEK293-10 cells were incubated with inhibitors and controls before the addition of exogenous IGF1 at 100 ng/mL. After 20 min in the presence (blue) or absence (red) of IGF1 at 100 ng/mL, the cells were lysed and phosphorylated AKT was measured by Western immunoblotting, as described in Materials and Methods. The insert provides a representative result with a band of the correct size (57 kDa) in each lane, as assessed by Western immunoblotting. All data are expressed relative to the level of AKT phosphorylation in the presence of 100 ng/mL IGF1. Identical letters indicate the absence of a significant effect, whereas different letters depict significant differences (*: *p* < 0.05; ****: *p* < 0.0001; the error bars indicate means ± SEM; *n* = 4; DMSO: dimethyl sulfoxide; kDa: kilodalton; all other abbreviations are explained in Figure 1).

To test the specificity of AKT phosphorylation in response to exogenous IGF1, HEK293-10 cells were treated with established inhibitors of IGF1 receptor signaling before the IGF1 incubation. The small molecule inhibitor BMS-754807 (BMS) disturbs the phosphorylation of IRS, and therefore blocks the IGF1 or insulin receptor signaling cascade. Because BMS has to be solubilized in DMSO, 0.1% DMSO in PBS was used as the BMS solvent control (Figure 3). Preincubation in BMS for 2 h significantly reduced the phosphorylation of AKT at the highest concentration (1 µM) as compared to the solvent control (*p* < 0.0001.) The inhibitor αIR3 antiserum also blocked the phosphorylation of AKT in response to exogenous IGF1 in our cellular system (*p* < 0.0001). The negative effects of BMS at the highest concentration were more prominent than the negative effect of αIR3 at the lower concentration of 100 ng/mL (*p* < 0.05). The inhibitors had no effect on the expression of total AKT.

#### 3.1.3. Pharmacokinetics of IGF-Dependent AKT Phosphorylation 

**Figure 4:**
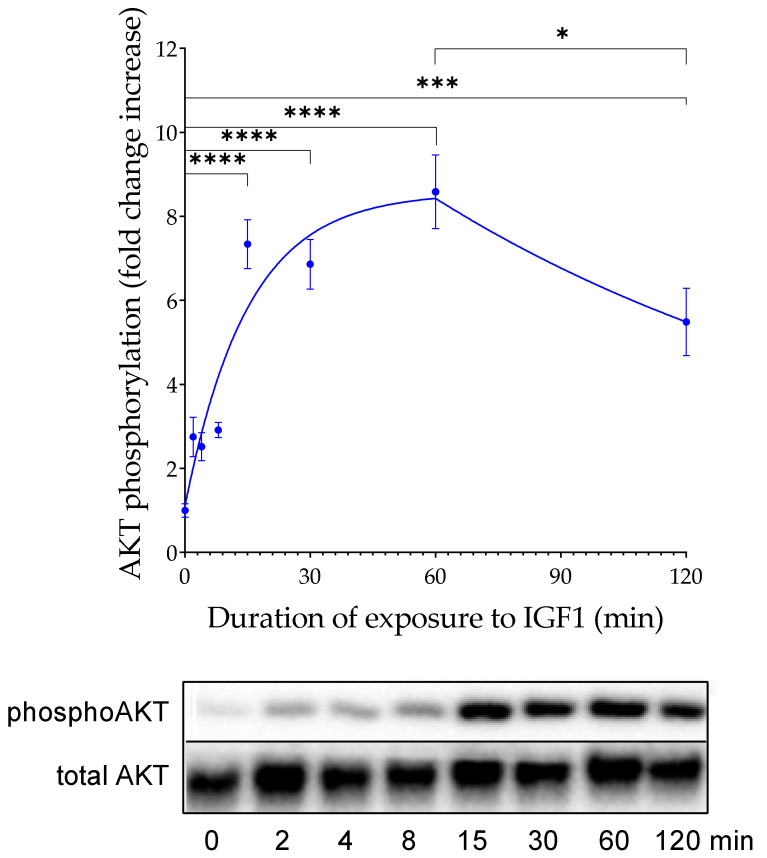
Temporal response of AKT phosphorylation after the addition of exogenous IGF1 at a concentration of 100 ng/mL to HEK293-10 cell monolayers. HEK293-10 cells were incubated for up to 120 min before cells were lysed and assayed for phosphorylated AKT. Only the significance levels of two consecutively time points are displayed (*n* = 3, means ± SEM, * *p* < 0.05, *** *p* < 0.001, **** *p* < 0.0001). The insert provides a representative result with a band of the correct size (57 kDa) in each lane, as assessed by Western immunoblotting for AKT phosphorylated at serine 473 (phosphoAKT) and for total AKT (abbreviations are explained in Figure 1).

In order to characterize the kinetics of IGF-dependent AKT phosphorylation in our cellular system, HEK293-10 cells were incubated with 100 ng/mL human recombinant IGF1 and AKT phosphorylation was assayed at eight different time points (Figure 4). A maximal response was detected 15 min after the start of the experiment (*p* < 0.0001). This is also the earliest time point when a significant effect could be fixed. At later time points, a further increase could not be observed, although phosphorylated AKT remained at elevated levels up to 60 min after the start of the experiment (*p* < 0.0001). At the latest time point, the signal intensity of phosphorylated AKT appeared to decline if compared to the previous time point, with the highest signal recorded at 60 min (60 min vs. 120 min: *p* < 0.05). Although the signal from the latest time point was no longer significantly different from the signal at 8 min (*p* = 0.0726), it was still higher than the signals at 2 min or 4 min (*p* < 0.05) and at 0 min (*p* < 0.001). Expression of total AKT was stable within the time frame tested. For the regression curve calculated in GraphPad Prism 9 (results are shown in Figure 4), the value *t*_0_ was set to 60 for the time point with the highest measured signal and the value *HoTNM* was set to 8.583 as the mean value for the signal at time point 60.

### 3.2. Preincubation with IGFBP2 Enhances IGF but Not Insulin-Dependent AKT Phosphorylation in a Dose-Dependent Manner

**Figure 5:**
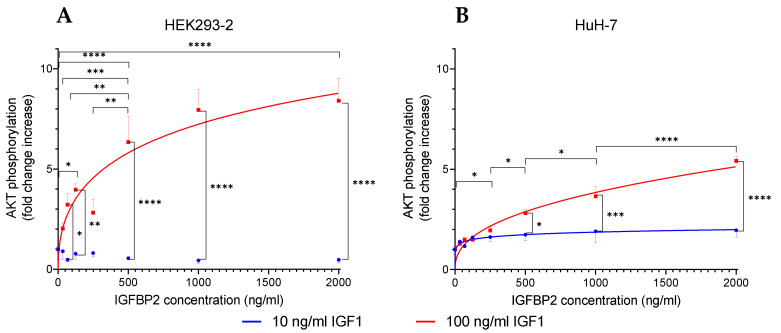
IGFBP2 increases IGF1-dependent phosphorylation of AKT in a dose-dependent manner. Monolayers of HEK293-2 cells (**A**) or HuH-7 cells (**B**) were preincubated for 24 h with different concentrations of IGFBP2 (0, 33.75, 67.5, 125, 250, 500, 1000, and 2000 ng/mL) in culture medium with 0.5% serum. For the bioassay, cell monolayers were incubated with 10 ng/mL IGF1 (blue) or 100 ng/mL IGF1 (red) for 20 min then lysed and assayed for phosphorylated AKT. Significance is provided for the comparison of different IGF1 or IGFBP2 concentrations (*n* = 3, means ± SEM, * *p* < 0.05, ** *p* < 0.01, *** *p* < 0.001, **** *p* < 0.0001). In one case, one outlier was identified by GraphPad Prism and not used for the calculation of the mean (*n* = 2 for HuH-7 cells preincubated in 2000 ng/mL IGFBP2 and challenged by 100 ng/mL IGF1; for abbreviations please also see Figure 1).

In vascular smooth muscle cells, it was demonstrated that IGFBP2 enhances IGF-related bioactivity [12]. We, therefore, questioned whether preincubation of IGFBP2 with HEK293 cells or other cell types also results in enhanced sensitivity. In addition, we questioned whether enhancement of IGF-dependent activation of AKT is dependent on the concentration of IGFBP2. Preincubation in IGFBP2 also resulted in massive induction of AKT phosphorylation in HEK293-2 cells (Figure 5A). Notably, IGFBP2 had a dose-dependent effect on IGF1-dependent AKT phosphorylation in HEK293-2 cells (Figure 5A). A significant increase of IGF1-dependent AKT phosphorylation was observed at concentrations up to 500 ng/mL. However, an enhancing effect of IGFBP2 was only observed at higher doses of IGF1 (100 ng/mL) in HEK293-2 cells. In contrast, even the highest concentrations of IGFBP2 had no impact on the effects of IGF1 on AKT phosphorylation at a concentration of 10 ng/mL. This finding is in principal agreement with the results presented in Figure 1, where an enhancing effect of IGFBP2 was observed at concentrations of 100 ng/mL or higher, but not at concentrations of 10 ng/mL or lower. The results further suggest that AKT phosphorylation is not an IGF-independent function of IGFBP2 in our cellular context. Preincubation with IGFBP2 did not enhance insulin-dependent AKT phosphorylation in 293-2 cells or total expression of total AKT.

We next questioned whether the BIRA assay could also be applied to other cell types. At concentrations of 250 ng/mL or above, HuH-7 cells were sensitive to the enhancing effects of IGFBP2 preincubation (Figure 5B). Compared to HEK293-2 cells, the response after 100 ng/mL IGF1 challenge appeared to persist at a lower level with significant increases, even at the highest dose of IGFBP2 preincubation and with 2000 ng/mL versus 1000 ng/mL IGFBP2 (*p* < 0.001). This indicates that saturation is not achieved by the 2000 ng/mL dose of IGFBP2, or in other words that IGFBP2 concentrations higher than 2 µg/mL may further increase IGF1-dependent AKT phosphorylation in HuH-7 cells. Again, an effect of IGFBP2 on IGF1-dependent AKT phosphorylation was not observed at an IGF1 concentration of 10 ng/mL. In our screening protocol for the study of effects of IGFBP2 preincubation, we also tested C2C12 and 3T3-L1 cells. Under the conditions of the present study, an enhancing effect was not observed in these latter two types of cells.

### 3.3. Induction of IGF-Related AKT Phosphorylation by Biological Matrices

In the second part of the study, different matrices (serum, cerebrospinal fluid, milk, and colostrum) were incubated with HEK293-10 cells for 20 min and phosphorylated AKT was assayed. 

#### 3.3.1. Age-Dependent Effects of Mouse Serum to Stimulate AKT Phosphorylation

**Figure 6:**
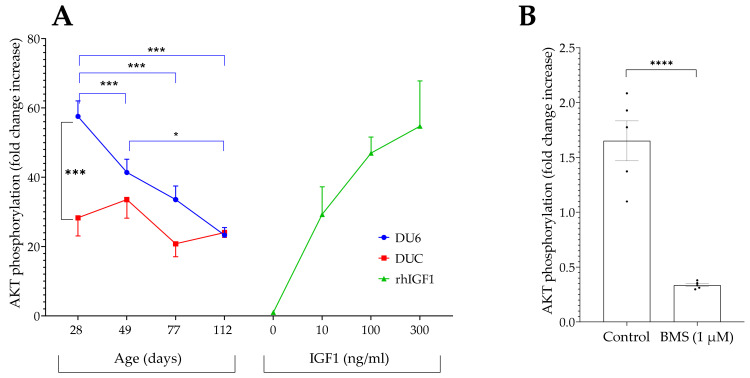
(**A**) Activation of AKT phosphorylation by diluted serum samples from male growth-selected mice (DU6) and unselected controls (DUC). Serum was prepared from mice between 28 and 112 days of age. Serum pools from five animals were diluted in PBS (1/5) and incubated for 20 min with HEK293-10 cell monolayers before lysis and assessment of AKT activation. Each pool was measured four times. In addition, AKT phosphorylation was assayed in the absence or presence of recombinant human IGF1 (rhIGF1; mean ± SEM; *n* = 4; * *p* < 0.05, *** *p* < 0.001). (**B**) The effect of BMS was tested in a diluted serum pool from 112-day-old DU6 animals. Therefore, HEK293-10 cell monolayers were incubated in EMEM containing 0.1% dimethyl sulfoxide (DMSO) as a control and with medium containing 1µM BMS-754807 and 0.1% DMSO (BMS) for 2 h before mouse serum was tested (mean ± SEM; *n* = 5; **** *p* < 0.0001; for abbreviations also see Figure 1).

At an age of 28 days, serum from male growth-selected mice (DU6) stimulated phosphorylation of AKT to a higher extent than serum from unselected controls (*p* < 0.001; Figure 6A). In serum from DU6 mice at this age, the ability of serum to induce AKT phosphorylation was compared to the activity of recombinant human IGF1 at concentrations of 100–300 ng/mL. By contrast, serum from age-matched unselected controls stimulated phosphorylation of AKT only at a level of 10 ng/mL rhIGF1. Between 28 and 112 days of age, a continuous decline of serum activity towards AKT phosphorylation was observed in male DU6 mice, but not in unselected controls. Accordingly, a significant reduction in the activity of male DU6 mouse serum was present at 49 vs. 28 days (*p* < 0.001) and at 112 vs. 49 days of age (*p* < 0.05). Preincubation of HEK293-10 cells with 1 µM BMS-754807 in EMEM and 0.1% DMSO for 2 h blocked almost 80% of the AKT phosphorylation when compared to preincubation of the cells in solvent control containing only 0.1% DMSO (Figure 6B; *p* < 0.0001). 

#### 3.3.2. Activation of AKT Phosphorylation by Serum from Individual Naked Mole Rats

**Figure 7:**
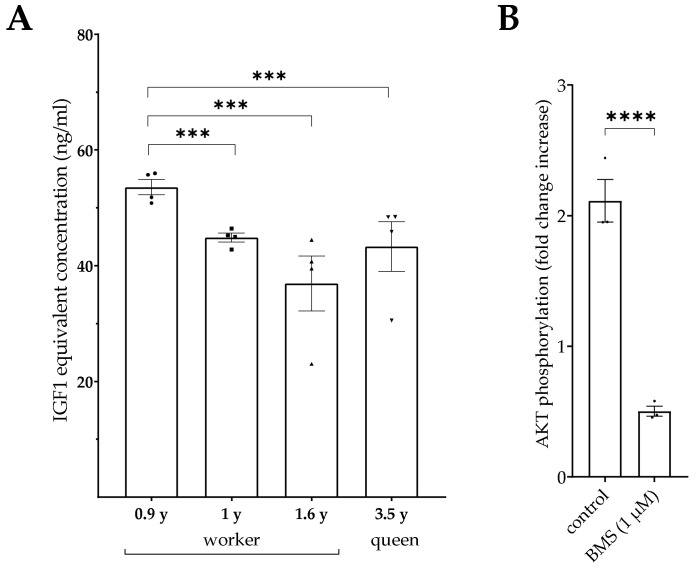
(**A**) Activation of AKT phosphorylation by diluted serum samples from female naked mole rats between 0.9 and 3.5 years of age, diluted in PBS and incubated for 20 min with HEK293-10 cell monolayers before lysis and assessment of AKT activation. After correction for dilution, IGF1 equivalency was calculated by interpolation using serial dilutions of IGF1. (**B**) The effect of BMS-754807 was tested in diluted serum pools generated from all naked mole rats. Therefore, HEK293-10 cell monolayers were incubated in medium containing 0.1% dimethyl sulfoxide (DMSO) as a control and with medium containing 1 µM BMS-754807 and 0.1% DMSO (BMS) for 2 h before the serum pools were applied (mean ± SEM; *n* = 3; *** *p* < 0.001; **** *p* < 0.0001; for abbreviations see Figure 1).

Serum samples from individual female naked mole rats were also diluted in PBS to a final concentration of 20% serum. Significantly higher bioactivity in the youngest worker (age 0.9 years) was found if compared to the older three animals (age 1 years to 3.5 years). Beside the age, there was no different bioactivity measurable, comparing the social status between the queen and the older worker (Figure 7A).

To test assay specificity for serum from naked mole rats, a pool was prepared using serum samples from four animals and added to cells previously incubated either for 2 h with 1 µM BMS-754807 in 0.1% DMSO EMEM or in 0.1% DMSO EMEM only. The results (Figure 7B) revealed inhibition of AKT phosphorylation by the small molecule inhibitor to ≈ 75% compared to AKT phosphorylation in cells incubated with naked mole rat serum without small molecule inhibitor (*p* < 0.0001).

#### 3.3.3. Activation of AKT Phosphorylation by Matrices of Human Origin 

We next tested IGF-related AKT phosphorylation in matrices of human origin in our cellular system. Because the samples were not available at quantities needed to perform multiple tests for technical replicates, pools were generated both for serum and for CSF from a total of ten different samples, respectively. 

**Figure 8:**
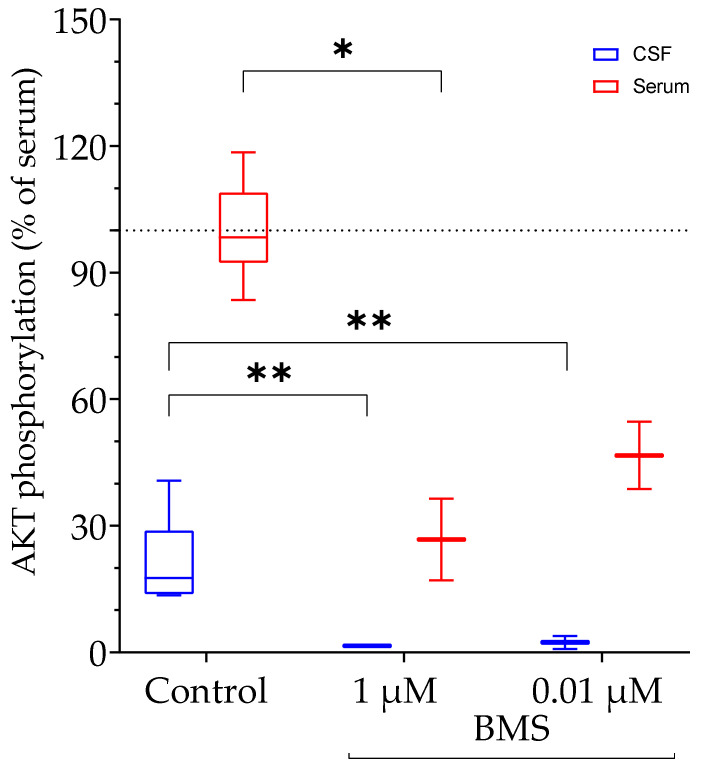
Activation of AKT phosphorylation by serum (red) and CSF (blue) samples of human origin. Diluted serum samples (1/5) pooled from ten human donors, whereas CSF was used without dilution. The pools were incubated for 20 min with HEK293-10 cell monolayers before lysis and assessment of AKT activation. In the graph, all data were corrected for their dilution factor. Each sample was measured six times. The dashed gray line corresponds to the mean of the serum (100%). In order to demonstrate the IGF dependency, BMS-754807 (BMS) was used to inhibit the IGF1 receptor prior to the incubation with human CSF or serum (*n* = 2; means ± SEM; * *p* < 0.05, ** *p* < 0.01; CSF: cerebrospinal fluid; for other abbreviations see Figure 1).

Human serum had a higher potential than CSF to induce phosphorylation of AKT in HEK293-10 cells (Figure 8). In both matrices, specific inhibition of IGF/insulin receptor signaling resulted in substantial reductions of AKT phosphorylation (serum: *p* < 0.05; CSF: *p* < 0.01). In human serum, BMS-mediated inhibition of AKT phosphorylation was ≈ 75%, and thus was similar to serum from naked mole rats. In human CSF, BMS at the same concentration blocked over 90% of AKT phosphorylation in HEK293-10 cells. 

#### 3.3.4. Activation of AKT Phosphorylation by Colostrum or Milk from Dairy Cows

The concentrations of IGFs and IGFBPs in dairy cow milk are highly dependent on the age of lactation. In order to study the potential activity of dairy milk for the activation of AKT, a longitudinal study was performed using colostrum or milk from animals at eight different lactation time points.

**Figure 9:**
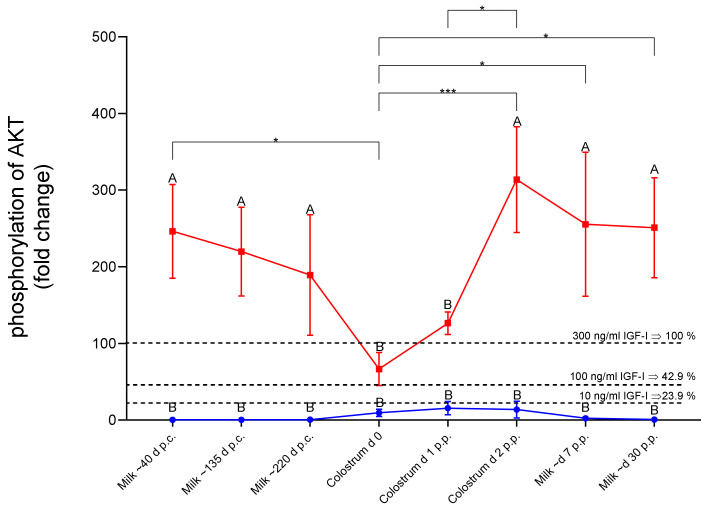
Phosphorylation of AKT-Ser473 in HEK293-10 cells by native (blue line) and acidified (red line) cow milk or colostrum samples during different stages during the lactation cycle. Cell monolayers were exposed to the samples for 20 min before cell lysis and assessment of AKT activation by Western immunoblotting. The dotted lines represent the levels of activation by 10, 100, and 300 ng/mL IGF1, respectively. Different letters indicate significant effects between native and acidified samples at the same time point (means ±SEM; *n* = 8; * *p* < 0.05, *** *p* < 0.001; p.c., post conceptionem; p.p., postpartum; for other abbreviations see Figure 1).

In contrast to native milk, native colostrum samples induced AKT phosphorylation in HEK293-10 cells equivalent to IGF1 at a concentration ≤ 10 ng/mL (Figure 9). Acidification resulted in a significant increase of AKT activation in both matrices (*p* < 0.001). The activity of acidified milk or late colostrum (lactation age: 2 days postpartum) was equivalent to IGF1 concentrations > 300 ng/mL. Notably, acidification had a stronger effect in milk than in colostrum at days 0 and 1 postpartum. Preincubation of HEK293-10 monolayers with BMS reduced the activity of acidified milk against AKT phosphorylation by 75% to 60%. Neutralization of acidified milk through the addition of NaOH at equimolar concentrations abolished the elevated potential of acidic milk towards AKT phosphorylation in HEK293-10 cells (Figure S2). Accordingly, activation of bioactivity in milk by acidification is reversible and can be controlled by pH.

## 4. Discussion

In biological matrices, the effects of the IGF system are controlled by peptide hormones and receptors, IGFBPs, IGFBP proteases, and inhibitors of IGFBP proteases [1]. In order to cope with the complexity of the IGF system, which has been explicitly discussed by the scientific community [18], and to describe the bioactivity of a given matrix in vitro, bioassays can provide specific information. In order to test the effects of IGF-related bioactivity on the phosphorylation of AKT in different biological matrices, we developed the BIRA assay, a robust and sensitive cell-based bioassay system. 

### 4.1. Short-Term Effects of Membrane-Bound IGFBP2 vs. Longer-Term Effects of Soluble IGFBP2

For decades it was unclear whether IGFBP2 has positive or negative effects on cell proliferation [19]. The molecular basis for IGFBP2 as a positive regulator of IGF1-dependent AKT-Ser473 phosphorylation was provided by the Clemmons lab [11,12]. Accordingly, we can distinguish the short-term effects of IGFBP2, which are related to cell surface interaction and which have positive effects on IGF-dependent signal transduction, from the longer-term effects of soluble IGFBP2, which are thought to block the interaction of IGF1 with IGF1 receptors. We questioned whether the short-term and positive effects of IGFBP2 on IGF-dependent signal transduction could be used to develop an assay to detect IGF-related bioactivity in signal transduction, characterized by improved sensitivity. We addressed this question using IGFBP2-transfected HEK293-10 cells, marked by elevated IGFBP2 secretion [13]. From our previous work we knew that HEK293 cells are sensitive to the effects of IGF1 [13], which was one of the premises for this study. In this cellular system, we studied in detail the longer-term negative effects of IGFBP2 on IGF-dependent cell proliferation. We demonstrated that with increasing concentrations of IGF1 or with LongR3 IGF1, lacking interaction with IGFBPs, cell proliferation can be rescued in IGFBP2-transfected HEK293-10 cells [13]. The duration of cell proliferation assays amounts to three to four days, whereas the effects of IGF-dependent AKT phosphorylation can be studied after a couple of minutes. Since the cells were washed before the assay, this period was not sufficient to establish inhibitory concentrations of soluble IGFBP2 in the extracellular space. First of all, HEK293-10 cells with high expression of IGFBP2 responded to a much stronger extent to exogenous IGF1 addition than transfected HEK293-2 cells, which do not express high amounts of IGFBP2. However, when HEK293-2 cells were incubated with conditioned medium from IGFBP2-transfected HEK293-10 cells or with different concentrations of recombinant IGFBP2, increased sensitivity towards high doses of exogenous IGF1 was established. Finally, the positive effects of recombinant IGFBP2 were not restricted to HEK293-2 cells, but were also identified in hepatic carcinoma cells (HuH-7). We thereby confirmed the mechanism originally described by the Clemmons lab in vascular smooth muscle cells [12]. Accordingly, a positive short-term effect of IGFBP2 on IGF-dependent cell signaling [11,12] was observed also in our cellular system. Notably, preincubation in recombinant human IGFBP2 did not enhance IGF-related AKT phosphorylation in 3T3 or C2C12 cells. At present, we can only speculate that coordinated AKT activation as described [11,12] may not be functional in all cell types as a rule. The longer-term inhibitory effects of soluble IGFBP2 (e.g., on cell proliferation [13]) can be neglected in the new bioassay. IGFBP effects as a function of the duration of IGFBP exposure were also discussed in vivo [20]. Notably, insulin-stimulated AKT phosphorylation at supraphysiological concentrations and preincubation with IGFBP2 did not affect insulin sensitivity in our system. 

### 4.2. Specificity

In order to define the specificity of IGF-related AKT phosphorylation, we blocked IGF1 receptors inside and outside the cells. To inhibit IGF1 interaction with the receptor outside the cells, we tested the effects of monoclonal antiserum (alpha IR-3) interacting with extracellular alpha subunits of the IGF1 receptor [21]. This interaction further blocks the binding of IGF1 to its receptor [22], and the antibody, therefore, has neutralizing functions for the IGF-dependent signal transduction through the IGF1 receptor. Incubation of IGFBP2-transfected HEK cells with alpha IR-3 antibody at both tested concentrations significantly inhibited AKT-Ser473 phosphorylation in response to IGF1. Additionally, intracellular inhibition of IGF1/insulin receptor signaling resulted in the substantial suppression of IGF1 I-dependent AKT phosphorylation. Intracellular inhibition of IGF1-dependent signal transduction was achieved using a small molecule (BMS-754807) that binds to the intracellular kinase domains from IGF1 and insulin receptors [23,24]. 

### 4.3. Time Dependency

To define the window for the readout of IGF-dependent AKT-Ser473 phosphorylation, we incubated IGFBP2-transfected HEK293-10 cells with 100 ng/mL IGF1 for up to 120 min and tested AKT phosphorylation at different time points. Maximal AKT phosphorylation was recorded 15 min after the addition of IGF1. The signal intensity remained elevated for an additional 45 min. Therefore, we decided to choose 20 min of IGF1 exposure before the readout of IGFBP2-enhanced AKT phosphorylation. Instead, the KIRA assay selected only 15 min of exposure to exogenous IGF1 before cell lysis [5,6]. This period was considered as a period short enough for the cellular system not to secrete IGFBPs, which may interfere with IGF1 in the supernatant [5,6]. From our results in a cellular system with the massive expression of IGFBP2, we had no indication of interference with secreted IGFBPs even until 60 min of exposure to IGF1. The reduction of IGF1-induced AKT phosphorylation 120 min after IGF1 application may be due to secreted IGFBPs or other secondary mechanisms, such as the decay of IGF1 or intracellular compensatory mechanisms to tune down the hormone response at the level of signal transduction. The phosphorylation of AKT in response to IGF1 is a complex event that includes binding of IGF to the IGF1 receptor, autophosphorylation of the IGF1 receptor, transfer of the signal to PI3K and PIP3, recruitment of AKT to the cell surface, and finally AKT phosphorylation at Ser437 by mTOR. In NIH3T3 mouse fibroblasts, phosphorylation of IGF1 receptors occurs within 13 to 25 s, whereas kinases located downstream of the IGF1 receptor are stimulated to their maximum 25–200 s after the addition of IGF1 [25]. This, on the one hand, may explain why the readout of IGF-induced phosphorylation at the level of canonical downstream kinases may require a longer exposure time compared to IGF1 receptor phosphorylation. However, this does not directly explain why a maximal response was achieved only 15 min after the IGF1 addition. Instead, the substantially longer lag time from application of IGF1 to maximal AKT phosphorylation may be related to the mechanisms of potentiation due to cell surface interaction of IGFBP2 [11,12]. According to the mechanism provided by Shen et al. [11,12], the control of AKT phosphorylation in response to IGF1 not only includes the canonical kinases described earlier, but additional kinases, phosphatases, substrates, and receptors [11]. AKT dephosphorylation is thereby inhibited by a mechanism involving IGF1 and IGFBP2, resulting in elevated levels of phosphorylated AKT. 

### 4.4. IGF-Related Bioactivity in Serum from Growth-Selected Mice and Controls and Its Age Dependency

The novel bioassay was used to analyze the biological activity in serum from growth-selected mice (DU6) and unselected controls (DUC) [26]. During the pubertal growth spurt in mice, we recently identified a higher molar ratio of IGF1 to the sum of IGFBPs in serum from DU6 mice compared to DUC mice [15]. Therefore, we postulated higher biological activity in giant DU6 versus control mice at the age of 28 days. In fact, we confirmed higher IGF-related biological activity in serum from DU6 mice at this age compared to controls. During the growth spurt at 28 days of age, the biological activity in serum from DU6 mice corresponded to more than 100 ng/mL of free IGF1 but less than 10 ng/mL in unselected controls. The different biological activities described here furthermore correlate with substantially higher concentrations of IGF1 in DU6 mice (≈1500 ng/mL) if compared to unselected controls (<1000 ng/mL) during the pubertal growth peak [15]. 

In addition, a second existing hypothesis was confirmed by the novel bioassay. In previous work, we described reduced ratios of IGF1 to the sum of IGFBPs in serum with increasing age [15]. Using serum from giant mice aged 28 to 112 days, we confirmed the prediction that biological activity is drastically reduced between the ages of 28 and 49 days in giant DU6 mice. At later time points, the IGF-related bioactivity is further decreased to levels according to <10 ng/mL of free IGF1. Additionally, in accordance with previous work [15], no decrease in IGF-related bioactivity was found in unselected control mice aged 28 to 112 days.

### 4.5. IGF-Related Bioactivity in Serum from Naked Mole Rats

The sequence homology of IGFs from naked mole rats amounts to more than 90% compared to their human or mouse orthologue [27]. Naked mole rats also express IGFBPs and PAPPA [27]. However, to the best of our knowledge, in naked mole rats IGF1 concentrations have not yet been tested by using existing diagnostic tools from other vertebrate species. Nevertheless, the IGF system is of great interest in this longest-living, and therefore unique, rodent animal model [28]. We used serum samples from individual animals and compared IGF-related bioactivity in serum samples from different worker animals to a serum sample from a long-lived queen. While we could not identify a general difference between serum samples from workers and the queen, we can use the bioassay to characterize IGF-related bioactivity in individual animals. Although it is difficult to obtain serum samples from naked mole rats, more samples should be used to set up a distinct study in this novel animal model in the future. Notably, the IGF-related bioactivity in samples from naked mole rats could directly be compared to other vertebrate species by using the novel bioassay to study the relative activity levels of the IGF system in different matrices and in different age groups.

### 4.6. IGF-Related Bioactivity in Serum and Cerebrospinal Fluid from Human Subjects

The growth, development, and functions of the brain are regulated by the coordinated regulation of local and systemic effects of the IGF system [29]. In CSF, IGF2 has been identified as a marker of mild cognitive impairment in Alzheimer’s patients [30] and as a biomarker for the progression of amyotrophic lateral sclerosis [31]. In CSF from patients with diabetic neuropathy, the biological activity of the IGF system is regulated by proteolytic degradation and the KIRA assay was used for determination of bioactive IGF in serum and CSF [10]. Interestingly, although IGF levels in CSF are less than 14% if compared to serum, bioactive IGF in CSF was ≈28% of bioactive IGF in serum, as determined by the KIRA assay [10]. We have also characterized the IGF system in serum and CSF samples, however from patients with multiple sclerosis [17]. We described the presence of IGFBP fragments in CSF and postulated regulation of IGF bioactivity by IGFBP proteolysis in this matrix. In our study, we used matched serum and CSF samples from patients. In these patients, the IGFs concentrations in CSF were less than 5% of the IGF concentrations in serum [17]. By use of the novel BIRA assay, we were able to identify IGF-related bioactivity at 20% of the level of serum. Accordingly, we confirmed published evidence [10] of the regulation of IGF-related bioactivity in CSF and provided evidence for the production of similar results using the new bioassay as compared to the well-established KIRA assay.

### 4.7. IGF-Related Bioactivity in Colostrum and Milk Samples from Dairy Cows

Mammary secretions are rich sources of IGFs and IGFBPs [32,33]. In particular, colostrum contains high concentrations of IGF1 [34]. In early breast milk, a serine protease was identified, which is responsible for the degradation of IGFBP2 [35]. Accordingly, in milk, the regulation of IGF-related bioactivity was discussed in the context of the effects on the mammary epithelia from the mother and the gastrointestinal tract from the suckling [35]. Additionally, in milk from dairy cows, the concentrations are high after calving and reduce towards mid and late lactation [36]. Therefore, Sejrsen et al. tested the biological activity of milk samples from different stages of lactation [36]. Similar to the protocol here, they selected mitogenic activity as the parameter for the readout of IGF-related bioactivity. In this study, higher mitogenic activity was identified in colostrum samples compared to milk from advanced lactational ages [36]. In addition, in this study, the interference of IGFBPs was suggested. In a subsequent study, the mitogenic potential of acidified or neutralized milk samples was also tested [37]. Since acidification may also activate the cytokine transforming growth factor beta (TGFB), the authors concluded that different factors might contribute to the biological activity of milk [37]. Similar to Purup et al., we also tested the effects of neutralization in milk samples after acidification. We directly applied acidic milk samples to our cell culture model, because after neutralization, free IGFs may return to their inactive IGFBP bound form. Very clearly, acidification correlated with enhanced biological activity in the form of AKT-Ser473 phosphorylation in the present study. We have no direct evidence that this activity is due to activation of TGFB for a number of reasons. First, inhibition by preincubation with BMS blocked phosphorylation of AKT by up to 75%. Second, neutralization of acidified milk also abolished the potential of milk to induce AKT phosphorylation. In contrast, activation of TGFB is not reversible. Therefore, we argue that during acidification, IGFs are released from their binding proteins, which may acutely induce the biological activity of IGFs. This mechanism could be involved during the development of the gastrointestinal tract in the growing suckling, at least as long as gastric proteolysis is not sufficient for the complete decomposition of peptides and proteins, and intact or fragmented peptides may arrive in the intestine in an active form. Since Elmlinger et al. have discussed the role of IGFBP proteases for growth and development of the intestinal tract [35], acidification may form a second mechanism controlling IGF-related bioactivity in milk. 

Interestingly, IGF-ELISAs also use acidification to release IGFs from their binding proteins before IGF determination. Accordingly, we may use the novel BIRA assay to estimate total IGFs or IGF-related bioactivity in species where no specific ELISA is available so far.

The BIRA assay is not dependent on IGFBP2-transfected HEK293 cells, since preincubation of HEK293 cells or Huh-7 cells for two hours in IGFBP2 is sufficient for increased sensitivity towards IGF-related AKT activation. The novel assay thus is not restricted to a certain cell type, although it is clear that not all cells, as a rule, express the required machinery to enhance the effects of the IGFs. Accordingly, the cellular response needs to be established in other cellular systems. Therefore, it is also required to determine the specificity of AKT phosphorylation in response to a given matrix. To do so, the small molecule inhibitor BMS can be used. With respect to different cell types, the effective doses of IGFBP2 used during preincubation to enhance the cells´ sensitivity to the effects of IGFs may be defined. So far, we have not yet tested the effects of IGF1 versus IGF2, which is an interesting issue for follow-up studies. 

## 5. Conclusions

In the present manuscript, we have developed and applied the BIRA assay as a novel bioassay to study IGF-related bioactivity in vitro. The assay is characterized by enhanced sensitivity and high specificity for the readout of IGF-related bioactivity. The test system enables the fast determination of IGF-dependent AKT-Ser473 phosphorylation, can partially be automated by the inclusion of the WES-technology, and does not depend on transfected cells or certain cell types, in general. The bioassay can be used to analyze IGF-related activity in different matrices from different species and can be adapted to study bioactive or total IGF. In addition, the BIRA assay can be used to assess the interactions between defined matrices and certain cell types. In particular, the BIRA assay serves as a novel method to study functional interactions between the matrix and cell type in relation to mTOR signaling as the major pathway of aging and survival.

## Figures and Tables

**Table 1:** Table of cell lines used and their respective media.

Cell Line	Passage Number	Cell Culture Media (Order Number, Distributor)
HEK293, HEK293-10, HEK293-2	2–30 15–25	EMEM (#BE12-611F, Lonza)
HuH-7	15–25	DMEM (#BE12-604F, Lonza)
C2C12	5–15	DMEM (#BE12-604F, Lonza)
3T3-L1	2–10	DMEM/F12 (#BE12-719F, Lonza)

## Data Availability

Data is contained within the article or Supplementary Material.

## Supplementary Materials

The following are available online at https://www.mdpi.com/2073-4409/10/3/482/s1: Figure S1: Dose-dependent increase of AKT phosphorylation in IGFBP2-transfected HEK293-10 cells and untransfected HEK293 cells. Figure S2: Effects of native, acidified, and neutralized milk samples on AKT phosphorylation in HEK293-10 cells. Table S1: Table of settings for WES analysis.
