# Supplementary material for: Development of a Sensitive Bioassay for the Analysis of IGF-Related Activation of AKT/mTOR Signaling in Biological Matrices
Source: Cells. 2021 Feb 24;10(3):482. doi: 10.3390/cells10030482 (PMC7995968; doi:10.3390/cells10030482)
Supplement: Supplementary file 1 [file cells-10-00482-s001.pdf]

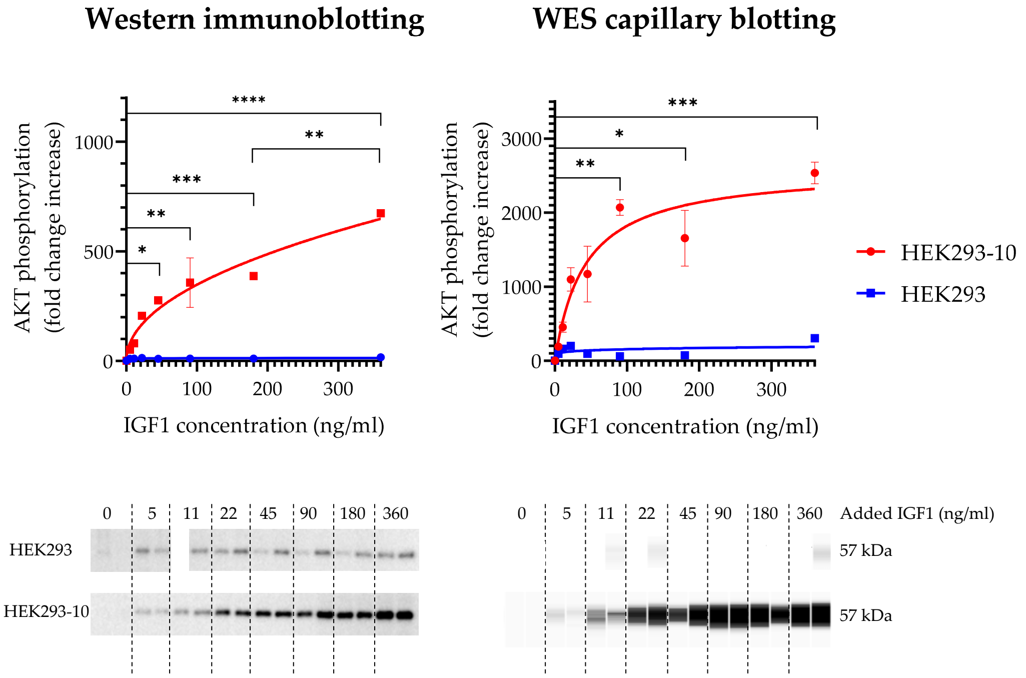

Scheme 1. Dose-dependent increase of AKT phosphorylation in IGFBP2-transfected HEK293-10 cells and untransfected HEK293 cells. Cells were incubated in different concentrations of human recombinant IGF1 as indicated for 20 min before they were lysed and assayed for phosphorylation of AKT by Western immunoblotting or capillary blotting (WES, Protein Simple) as described in Materials and Methods (mean ± SEM; n = 23, * p < 0.05, ** p < 0.01, *** p < 0.001, **** p < 0.0001).

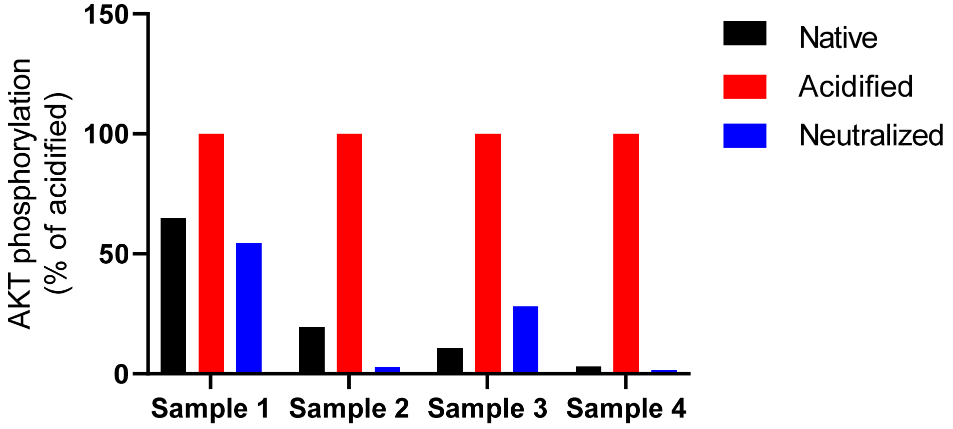

Scheme 2. Effects of native, acidified, and neutralized milk samples on AKT phosphorylation in HEK293-10 cells. Samples from four individual dairy cows were incubated with HEK293-10 cell monolayer for 30 min, and AKT phosphorylation was assessed by Western immunoblotting.

Scheme 1. Table of settings for WES analysis.

Menu	Setting	Value
Peaks	Molecular Weight	58 kDa
	Range	± 15 %
Range	Minimum	1.0
	Maximum	250.0
Baseline	Threshold	10.0
	Window	25.0
	Stiffness	1.0
Peak Find	Threshold	20.0
	Width	12.0
	Area Calculation	Gaussian fit
Advanced	Peak Width	15
	Allowable Drift	100
	Peak Fit Starting Width Ratio	0.5
	Median Filter Threshold Ratio	0.1
	Median Filter Threshold Limit	10
